# Repercussions of sickle cell disease and sickle cell ulcers for men inserted in the world of work

**DOI:** 10.1590/1980-220X-REEUSP-2022-0384en

**Published:** 2023-04-28

**Authors:** Dayse Carvalho do Nascimento, Gleysson Coutinho Santos, Samira Silva Santos Soares, Sheila Nascimento Pereira de Farias, Luana Ramos Garcia, Luana dos Santos Cunha de Lima, Pedro Miguel Santos Dinis Parreira, Norma Valéria Dantas de Oliveira Souza

**Affiliations:** 1 Universidade do Estado do Rio de Janeiro, Faculdade de Enfermagem, Rio de Janeiro, RJ, Brasil.; 2 Universidade Federal do Rio de Janeiro, Escola de Enfermagem Anna Nery, Rio de Janeiro, RJ, Brasil.; 3 Universidade de Coimbra, Faculdade de Enfermagem, Coimbra, Portugal.

**Keywords:** Anemia, Sickle Cell, Leg Ulcer, Work, Men, Enterostomal Therapy, Anemia de Células Falciformes, Úlcera de la Pierna, Trabajo, Hombres, Estomaterapia, Anemia Falciforme, Úlcera da Perna, Trabalho, Homens, Estomaterapia

## Abstract

**Objective:**

To analyze the repercussions of sickle cell disease and sickle cell ulcer for men in the world of work and discuss the challenges faced to remain in the work environment.

**Method:**

A qualitative study, developed at the dressing clinic and at a stomatherapy clinic. Twenty men with sickle cell disease and sickle cell ulcer participated, applying a semi-structured interview script. The software Interface de R pour les Analyses Multidimensionnelles de Textes et de Questionnaires was used for treatment and lexical data analysis.

**Results:**

The Descending Hierarchical Classification enabled the creation of classes: Man with sickle cell disease and sickle cell ulcer: experiences and repercussions; and Coping measures adopted by men with sickle cell disease and sickle cell ulcer to stay at work.

**Conclusion:**

Disease and injury repercussions involve biopsychosocial dimensions, highlighting the need for professional training to assist with competence and humanity. Strategies adopted to maintain work are breaks in the working day, use of analgesics to relieve pain, allocating time during work to apply dressings.

## RESUMO

**Objetivo:**

Analisar as repercussões da doença falciforme e da úlcera falcêmica para homens que atuam no mundo do trabalho e discutir os desafios enfrentados para se manterem no ambiente laboral.

**Método:**

Estudo qualitativo, desenvolvido no ambulatório de curativo e em uma clínica de estomaterapia. Participaram 20 homens com doença falciforme e úlcera falcêmica, aplicando-se roteiro de entrevista semiestruturado. Utilizou-se o *software Interface de R pour les Analyses Multidimensionnelles de Textes et de Questionnaires*, para tratamento e análise lexical dos dados.

**Resultados:**

A Classificação Hierárquica Descendente possibilitou a criação das classes: *Homem com doença falciforme e úlcera falcêmica: vivências e repercussões*; e *Medidas de enfrentamento adotadas pelo homem com doença falciforme e úlcera falcêmica para manutenção no trabalho*.

**Conclusão:**

As repercussões da doença e lesão envolvem as dimensões biopsicossociais, evidenciando a necessidade de capacitação dos profissionais para assistir com competência e humanidade. As estratégias adotadas para manutenção no trabalho são pausas na jornada laboral, uso de analgésicos para aplacar a dor, destinação de tempo durante o trabalho para realização dos curativos.

## RESUMEN

**Objetivo:**

Analizar las repercusiones de la drepanocitosis y la drepanocitosis para hombres que actúan en el mundo del trabajo y discutir los desafíos enfrentados para permanecer en el ambiente de trabajo.

**Método:**

Estudio cualitativo, desarrollado en la clínica de vendajes y en una clínica de estomaterapia. Participaron 20 hombres con drepanocitosis y drepanocitosis, aplicando un guión de entrevista semiestructurada. Para el tratamiento y análisis léxico de los datos se utilizó el software Interface de R pour les Analyses Multidimensionnelles de Textes et de Questionnaires.

**Resultados:**

La Clasificación Jerárquica Descendente permitió la creación de clases: Hombre con anemia falciforme y úlcera falciforme: experiencias y repercusiones; y Medidas de afrontamiento adoptadas por el hombre con enfermedad de células falciformes y úlcera falciforme para permanecer en el trabajo.

**Conclusión:**

las repercusiones de la enfermedad y lesión involucran las dimensiones biopsicosociales, destacando la necesidad de formación profesional para asistir con competencia y humanidad. Las estrategias adoptadas para mantener el trabajo son pausas en la jornada laboral, uso de analgésicos para aliviar el dolor, asignación de tiempo durante el trabajo para aplicar curas.

## INTRODUCTION

The experience of illness is built through social and cultural processes experienced by individuals, as well as through the experience of suffering imposed on each human being’s life, the physical symptoms experienced, the meaning of the disease for people, in coping or in line with care practices^(1)^. In this regard, this article listed as an object of study the repercussions of sickle cell disease and sickle cell ulcer (SCU) for men active in the world of work.

The choice to focus this study on the world of work is based on the fact that work occupies a central role in people’s lives; therefore, it has impacts when it is absent, or in the experience of pleasant work experiences or not, especially considering the process of illness.

Sickle cell disease (SCD) is characterized by a set of chronic diseases with an autosomal recessive inheritance pattern. The blood cell called red blood cell, responsible for transporting oxygen in the bloodstream, is modified and its conformation becomes sickle or half moon. Sickled red blood cells lose flexibility and become stiff, which negatively impacts organ oxygenation, causing a phenomenon known as vaso-occlusion^(2)^.

SCD generates a severe and degenerative condition, with multiple damages, often irreversible, which, without follow-up, can present high morbidity and mortality, both in childhood and in adulthood, triggering cardiopulmonary and renal complications, bone necrosis, eye lesions, leg ulcers, among others repercussions^(3)^.

Given the severity of the disease, the Ministry of Health, through Ordinance 1,391/2005, created the Brazilian National Policy for Comprehensive Care for People with Sickle Cell Disease (PNAIPSCD – *Política Nacional de Atenção Integral* às *Pessoas com Doença Falciforme*), aiming at reducing the high rates of morbidity and mortality and of individuals with hemolytic disease chronic, with a focus on intercurrence prevention and treatment, patient education, genetic counseling, multidisciplinary care and access to all levels of care^(4)^.

From a gender perspective, it appears that, between men and women, the male population is more vulnerable to the disease, with earlier deaths, as a result of greater exposure to behavioral and cultural risk factors, constructed from male stereotypes, especially in the young adult stage^(5)^. Thus, men are not allowed to show vulnerabilities and weaknesses, which delays or prevents the search for care units for health promotion, disease prevention, cure and rehabilitation. Moreover, they still remain as household providers and, therefore, need to maintain their jobs to guarantee personal and family subsistence, negatively impacting the investment in maintaining their own health^(6)^.

As previously highlighted, SCD can cause injuries to the lower limbs resulting from blood vessel occlusion at the site, which are vulnerable to trauma and can cause wounds that are difficult to heal. These injuries require care, such as replacement use, attendance at consultations, dressing, medication use, which affect carrying out work, leisure, sexuality, self-image activities, among other aspects of a day in the life of people affected by SCD and SCU^(7)^.

Thus, SCD is manifested by the presence of SCU, physical discomfort, loss of muscle strength, weakness, hemolytic anemia, frequent pain crises, recurrent infections and stroke. However, in men, there is another complication, the presence of priapism, characterized by involuntary and intensely painful genital erection that, if not effectively cared for, can lead to erectile dysfunction or even mutilation of the genital organ^(8)^.

Furthermore, ulcerative lesions may recur and be followed by painful crises, intense fatigue, priapism, presence of odor and high exudation resulting from the wound, predisposition to infections, difficult healing. Furthermore, due to the stereotype imputed to the male figure of invulnerability, the man has significant psychophysical and social suffering, with repercussions in social life and in their performance in the world of work^(8)^.

It is noteworthy that SCU recurrence will depend on the adoption of measures for self-care, productive activity that individuals develop, characteristics of life habits, family support and the local health network, among others. A study addresses that the prevalence and incidence of SCU involve geographic characteristics, age group and SCD evolution. Thus, it is inferred that the prevalence is 18.6% in Ghana, 3.5% in Italy and 2.4% in the United States^(9)^.

From the perspective of work, it is emphasized that it occupies a central role in individuals’ lives, as it is through it that human beings guarantee material subsistence. However, work goes beyond materiality, it imprints subjective aspects on the human being that affects the feeling of usefulness, belonging to a group, social status, pleasure or suffering in the face of the development of a work activity, giving meaning to human being’s life^(10)^.

From this perspective, the meaning of work for men with SCD and SCU transits between something positive, when they contribute to providing for the family to reinforce the male role in society, to make them participate in society, passing through negative feelings, when they feel pain, shame, impotence, frustration, tiredness resulting from the disease and injury situation, but need to remain at work. Finally, the work is dialectical, relevant and not neutral in relation to the subjectivity of these men^(11)^.

By considering this problem, this study aimed to analyze the repercussions of SCD and SCU for men active in the world of work and discuss the challenges faced in order to remain in the work environment.

## METHODS

### Study Design

Descriptive-exploratory research, with a qualitative approach. It was based on the Consolidated criteria for REporting Qualitative research (COREQ) script to describe the method^(12)^.

### Local

Two fields were used for the research: a dressing clinic in a large hospital and a nursing clinic in stomatherapy, allocated in a specialized polyclinic, both linked to the health complex of a public university located in the city of Rio de Janeiro. January, Brazil. The choice of these locations for develoing the study was based on the specificity of care for people with wounds of different etiologies, in particular sickle cell wounds.

### Population

Participants were 20 men with SCU, who were followed up in the aforementioned fields, 18 at the dressing outpatient clinic and two at the stomatherapy clinic, with the eligibility criteria being verified and following the principle of saturation.

### Selection Criteria

Inclusion criteria of participants were men of working age who developed a SCU injury while working. Exclusion criteria were men with some cognitive deficit and those who had permanent limitations for work.

### Data Collection

Data collection was carried out between August and November 2019, through a semi-structured interview, with an average duration of 60 minutes. Upon recording the medical records and the inclusion criteria, study participants were contacted by telephone and invited to contribute to the research. After accepting to participate in the study, the day, time and place for the interview were scheduled, according to participants’ preference.

An interview script was used with 26 questions, of which 22 characterized the men interviewed and the others related to the object of study, namely: i) talk about what it is like to have a sore leg for a long time; ii) talk about the repercussions of the wound for your work; iii) talk about how you perceive your work in your life; iv) talk about how health professionals, especially nurses, could help with your illness.

A pre-test was carried out on a small sample (four men), in order to identify the need for adjustments in the writing and understanding of addressed items. It was not necessary to change the instrument and the interviews were incorporated into the total study sample.

### Data Analysis and Treatment

For the treatment of collected data, the software *Interface de R pour les Analyses Multidimensionnelles de Textes et de Questionnaires* (Iramuteq), version 7.2, was used. The use of this software in Brazilian qualitative research has grown in recent years and this is justified by the growing movement towards the use of tools/applications to support qualitative research, expanding methodological rigor. Furthermore, Iramuteq is a free and freely accessible program^(13)^.

Iramuteq has five possibilities for data processing: i) statistical (lexicographic) analyses; ii) specificity and factorial correspondence analysis; iii) Descending Hierarchical Classification (DHC); iv) similarity analysis; and v) word cloud.

For the purposes of this study, it was decided initially to use analysis through DHC, as it provides a more robust data appreciation and is the most used method in health research that uses Iramuteq. Subsequently, the word cloud was processed, which, although simpler, is visually interesting and easy to understand. The DHC separated the corpus (material resulting from the interviews) into classes and allowed recovering the text segments (significant excerpts from the interviewees) for subsequent data interpretative and assertive analysis, distinguishing the content of one class from another^(13)^.

The word cloud referring to each class brought a graphic representation of the words according to the frequency of appearance in the texts. Thus, the larger words were the ones that appeared more often in the analyzed classes, and the smaller ones, with less frequency.

### Ethical Aspects

The study was approved by the Research Ethics Committee (REC) of the *Universidade do Estado do Rio de Janeiro* (UERJ), in 2019, under Opinion number 3,292,609. The principles and norms pre-established by Resolution 466/2012 of the Brazilian National Health Council (CNS) are followed, which regulates research involving human beings. Anonymity was guaranteed to participants, who were cited by a code (e.g., E1, E2… E20) generated after the consent process, through the Informed Consent Form (ICF).

## RESULTS

The study included 20 (100%) men with SCD and SCU, with a mean age of 42.25 years and mostly married (n = 12/60%). Moreover, 13 self-declared black (65%); 17 had a family income of one to three minimum wages (85%); all (100%) claimed to have a belief in a religion; and 11 (55%) declared to have incomplete primary education. Still, with regard to housing, all (100%) lived with family or friends, evidencing the possibility of having help from the people who cohabited with participants. When asked about their working life, 18 (90%) participants reported working informally, even though six (30%) were retired, and two (10%) declared receiving a Continuous Cash Benefit Program (BPC – *Benefício de Prestação Continuada*).

From DHC, two classes were originated: i) *Man with sickle cell disease and sickle cell ulcer: experiences and repercussions*; and ii) *Coping measures adopted by the man with sickle cell disease and sickle cell ulcer to stay at work*.

### Man with Sickle Cell Disease and Sickle Cell Ulcer: Experiences and Repercussions

**Figure 1.**
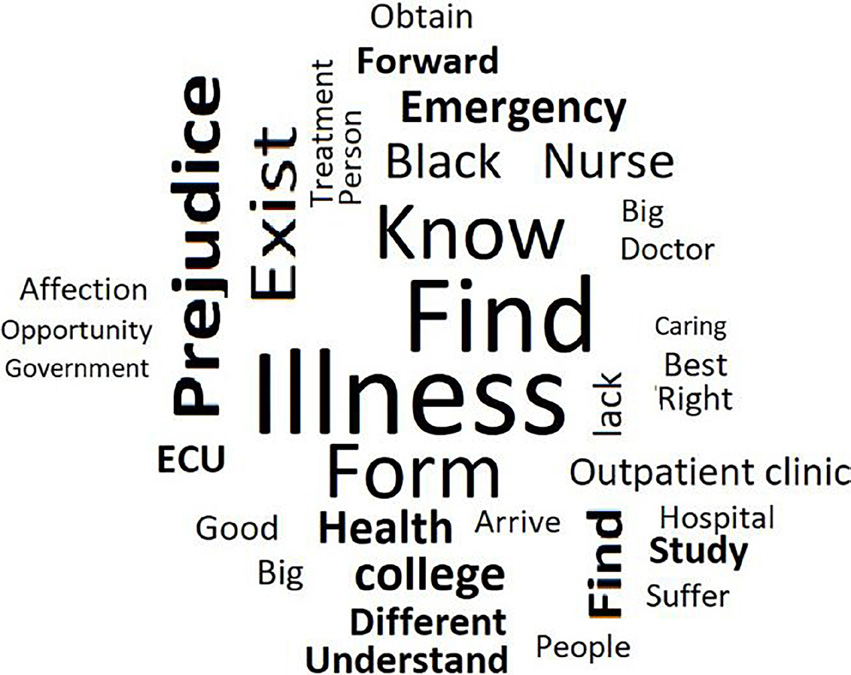
Word cloud about the man with sickle cell disease and sickle cell ulcer: experiences and repercussions (n = 20) – Rio de Janeiro, RJ, Brazil, 2019.

The most frequent words in this class were: disease; meet; form; prejudice; exist; to know; nurse; black; emergency. In order to highlight the highlighted words, the cloud of words resulting from this class is presented below (Figure 1).

SCD, since it is chronic in nature, with late diagnosis, leads individuals to the need to be followed by health professionals to carry out consultations and routine exams, successive hospitalizations, family and social distancing. This situation is exemplified from the text segments highlighted below:

*(…) I have sickle cell anemia. I know that I will always need help, because my illness is forever. I will not get cured! I get better and worse, all the time! Hospitalizations and readmissions. Also, that wound is damned, but it’s because of anemia (E7).*

*(…) I suffered a lot, because my diagnosis came too late… I went to several emergencies… I had many hospitalizations until I found out and got the treatment right. This had repercussions for my life as a whole (E5).*

Another aspect cited by participants was the lack of scientific technical preparation of the health team to treat people with sickle cell anemia, especially in emergency services, in recognizing the clinical symptoms of the disease and in treating SCU. Due to this situation, the participants chose to abstain from emergency care.

*Many physicians at the ECU (Emergency Care Unit), nurses and other professionals do not know what it is, and if they do not know what it is, they do not know how to treat it. You have to have training, a subject just about this disease in the college curriculum. They only know in specific hospitals, you call them reference hospitals, and these are normally far from our homes, but I prefer distance to not being treated properly (E12).*

*I’ve had a very bad time because no one knows how to treat me. My illness is different. There’s so much in the world, and many still don’t know it right. I try to go only to the hospital that knows me, that knows SCD. Is it far from my house? Yes, it is, but I prefer to go there, because I run away from any emergency or hospital that doesn’t know how to treat me (E10).*

Another result captured was that professionals centered care on the disease and drug treatment, neglecting care with SCU.

*Worst of all, the professionals only talk about the disease, about medications in general, and they don’t look at the wound, no one cares about the wound, and it’s something that doesn’t heal (E19).*

The existence of labels related to the wound by the nursing team in relation to men with SCD was also verified, verifying that professionals often discredit the disease’s pain and other repercussions. This result can be evidenced in the text segments presented below.

*It is very difficult to depend on a professional who does not know what he is doing. I’ve heard a professional say that I was exaggerating my pain (E06).*

*I take pain medication; I avoid hurting myself. Hospital I only go as a last resort, because they seem to think it’s freshness. The problem is that, when I go, the disease has already decompensated and things get ugly for me (E11).*

Furthermore, the racial issue emerged in this class, evidencing the historical process of SCD with the Afro-descendant population.

*(…) here in Brazil, prejudiced country that it is, racist that it is, a person having a disease and being black, having hurt is not easy. They don’t even look at us! (E11).*

*Black, poor and sick suffer more in Brazil, I feel it in my skin. It is more difficult for us to take care of the sickle cell wound and work, because it is expensive to have a wound, a lot of expense (E19).*

### Coping Measures Adopted by the Man with Sickle Cell Disease and Sickle Cell Ulcer to Stay at Work

**Figure 2.**
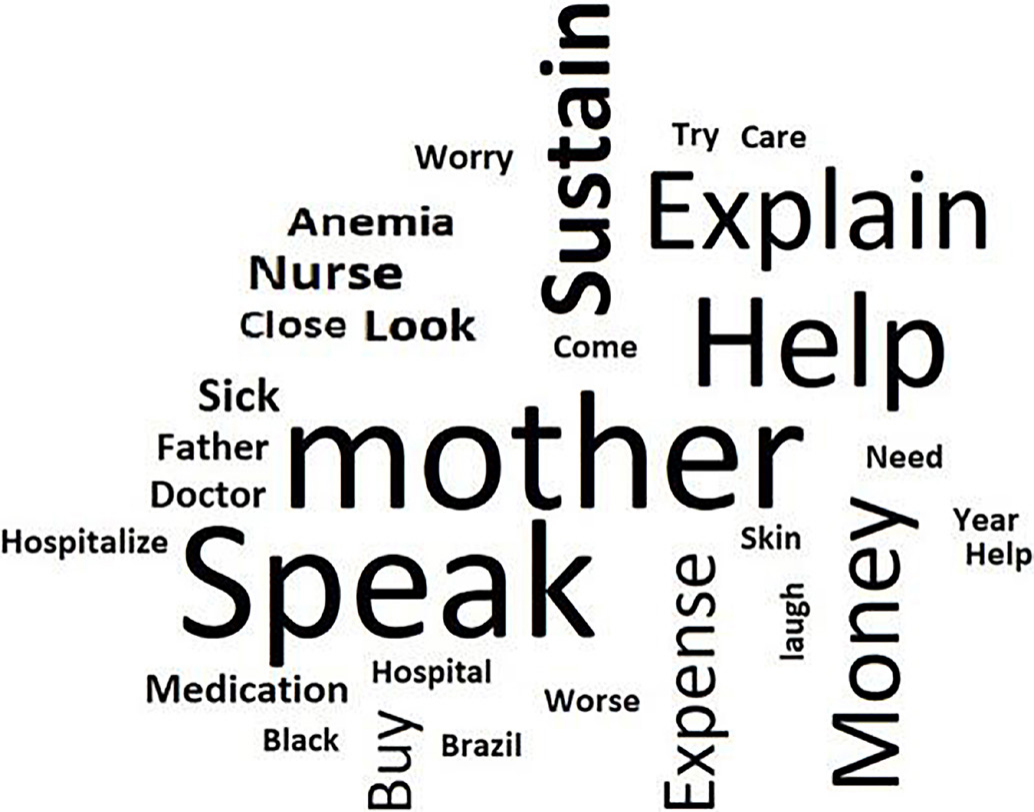
Word cloud about coping measures adopted by men with sickle cell disease and sickle cell ulcer to stay at work (n = 20) – Rio de Janeiro, RJ, Brazil, 2019.

In this class, the segments of texts in which the most frequent words were: mother; speak; help; explain; money; and provide. In this way, these words and the analysis of the text segments expressed the coping measures that men with SCU adopted to stay at work, in addition to dealing with family and social supports that help them in material survival and minimization of suffering resulting from the disease. The word cloud below shows these words (Figure 2).

SCU exposes individuals to a situation of vulnerability, being observed in the subsequent text segments that socioeconomic conditions reflect on the basic human needs of these individuals, negatively influencing the disease evolution and making treatment difficult.

*I’ve already borrowed money, I’ve already sold my things to buy medication, because, often, there’s no one there on Rua México… it’s like food, I can’t do without my medication, because if that happens, my blood gets thick and I have a severe pain attack (E10).*

*I spent three months without earning a penny. I went crazy! Then, I learned that you shouldn’t take too long to ask for help (E1).*

It is observed that family role, especially mothers, is essential in the preventive and evolutionary process of the disease, as it represents financial and emotional support so that men comply with treatment, strengthen self-esteem and learn self-care practices.

*I know I can always count on you to help me with my mother and with my God. I always pray to have the strength to keep fighting for life (E20).*

*I have a lot of expenses, it’s medicine, it’s bandage, it’s food. If I don’t have medicine, it makes up for my illness. It is a testament to poverty indeed. Is obvious! Then my wound gets worse, but I need to keep working, even with my mother’s help (E5).*

Faced with the costs generated by treatment and the responsibility for supporting the family, it appears that men with SCD and SCU find coping measures to remain in the world of work and be productive.

*I have to support the house. I take care of myself in my own way. I have a family that depends on me (E15).*

*In the middle of the day, I find a way to put my legs up, change the bandage and endure everything because it depends on my salary and so does my family! (E4).*

*You know that sometimes I forget even the pain of the wound. I take medication to solve it. I do my dressing properly and go to work at work (E19).*

From participants’ perspective, nurses provide guidance on care for SCU, allowing them to create strategies in the work environment to perform dressings and remain in their jobs, carrying out work activities without prejudice to the organization of work.

*I don’t miss my appointments so I don’t have problems, I take my medication correctly. I follow what the nurses teach me to perform the dressing, because I still want and need to work a lot (E15).*

*I’ve been hospitalized several times. But, in my work, I try to solve my dressing alone, when possible, with the guidelines I receive from the nurses (E19).*

Another relevant issue was religion as an inner strengthening strategy to face the changes and complications resulting from the disease, mainly to deal with the need to stay at work, as evidenced in the text segments presented below.

*I pray and ask that I have the strength to continue working. The disease makes people look at me differently at work, but I pretend I don’t notice and so I do my part. That’s my strategy (E10).*

Jesus knows the difficulty I have in dealing with the disease, my family’s life and my work. But being a man means having to answer for everything. I pray for strength and health! (E20).

## DISCUSSION

As it is an incurable chronic disease with multisystemic disorders that generate psychophysical and social suffering, SCD treatment is carried out throughout life and, for it to be successful, patients and their families must be aware of the signs and symptoms and adopt measures to act in advance in the various intercurrences^(11,14)^.

As highlighted, lifestyle and health conditions, which are associated with socioeconomic, political and cultural factors, significantly reflect and affect the lives of men with SCD, since this group is the most vulnerable to diseases, above all, to chronic and serious illnesses.

Therefore, it is noted that, in the face of hegemonic masculinity, strength, virility and invulnerability, attributes that are imposed on men, by a social ideology of the centralizing masculine, dominant and fearless in the face of risks and dangers, the male population has a significant impact of the disease. Thus, men tend to neglect the disease, are reluctant to seek help and discuss their own health, due to feelings of fear, shame, carelessness, impatience, in the face of other life priorities, thus making it difficult to change lifestyle habits and presenting decompensation of the pathological condition^(10)^.

In this research, it deserves attention the fact that men only seek health services at the extreme moment, which generates the need for successive hospitalizations. A study demonstrates that men seek self-treatment or alternative conducts in situations that trigger crises or when they perceive some abnormality, and only go to the health service in extreme cases^(15)^.

Thus, it is up to health professionals to qualify and develop strategies on changes in the welcoming and implementation of adequate care, directed to the problems of this population, based on the understanding of the needs of individuals with SCD, since the shortage of men in health care units health cannot be considered irresponsible, but justified by the role imposed on the male population^(16)^.

Brazil is one of the pioneering countries in establishing men’s health, through the Brazilian National Policy for Comprehensive Care for Men’s Health (PNAISH – *Política Nacional de Atenção Integral à Saúde do Homem*), launched in 2009 by the Ministry of Health (MoH). This program aims to promote health actions that make it possible to understand the reality of men in the age group between 20 and 59 years old, with a range of socio- cultural and political-economic scenarios. In this perspective, the importance of this policy is perceived to help in prevention and men’s compliance in SCD treatment^(17)^.

Thus, it is understood that SCD in men has specificities, as it hinders their participation in the world of work, weakening the role of home provider and autonomous and invulnerable man. It also interferes with subjectivity, as it distorts body image, resulting in changes in clothing, interpersonal relationships, leisure, life projects and sexuality^(18,19)^.

Another relevant aspect was related to health professionals’ unpreparedness in the recognition of clinical signs and symptoms and in SCD treatment, confirming the lack of knowledge and lack of information about the disease and treatment, especially in emergency units. Because of this, patients are often placed in a situation of vulnerability and embarrassment, reducing sick people to stereotypes, giving them discourteous and unequal treatment, discouraging the need to seek help, when necessary, from these services. Thus, there is damage to therapy, since emergency services are closer to homes, compared to reference locations^(20)^.

Thus, it is necessary to train health professionals to understand the pathophysiology that involves the disease and to promote humanized care, with a holistic and multidisciplinary approach, to reduce the physical, social and psychological impacts resulting from a chronic and lasting illness. Moreover, SCU treatment is slow and takes a long time to heal, with high rates of recurrences. Therefore, to treat it, health professionals must base themselves on scientific evidence and develop technical skills that guide conduct^(19)^.

The centrality of professionals in diagnosing the disease and in medicalization also emerged from the results. This situation raises fragility in the communication between a health professional and a person with SCD, making it difficult to provide comprehensive care for individuals, with little emphasis on their biopsychosocial needs in this context.

Many health professionals, unaware of the course of the disease and its treatment, minimize pain-related suffering, either because patients constantly complain of pain and frequently request medication, or because they believe that people with SCD are addicted to analgesics. and opioids, because these medications have the risk of dependence as a side effect. This generates fear and restriction by professionals in prescribing and administering medications. As a consequence, patients feel labeled as addicts and perceive that the health team is intolerant of them, responding to them harshly, leading to an ineffective and resolving relationship^(21)^.

As these professionals minimize the suffering related to pain and do not overlook the painfulness caused by SCU, men end up not complying with treatment and, on their own, perform dressings and use analgesics in underdoses or ineffective doses, in an attempt to alleviate the pain resulting from the disease and SCU^(22)^.

It is noteworthy that, even with the presence of a chronic wound that generates pain, odor, extravasation of exudate into clothing, heaviness in the legs and tiredness, feelings of fear, anguish and insecurity that, for many times, make it impossible to carry out activities^(23)^, men omit these repercussions and find coping measures to maintain the employment relationship, as they need it to survive and ensure autonomy. In this way, it guarantees family support and eases the suffering that the disease imposes.

The conveyed knowledge that the black population is more vulnerable to certain diseases is often the result of practices of racism. It is with these inconsistencies that power relations are constituted and disseminated based on biologicist arguments and conceptions, and it is from these contexts that the picture of racial inequities in health is maintained^(24,25)^.

Studies have found that health professionals believe that black individuals are more resistant to pain, and people with sickle cell anemia are capable of withstanding it without the need for analgesia^(25,26)^. The weakness in the scientific basis and the presence of racial prejudice imply an association between drug addiction and an individual’s skin color on the part of the health team^(25)^.

In this regard, this prejudiced thinking causes professionals not to provide adequate treatment for pain, showing that, at various times, these professionals are so immersed in the logic of prejudice and racial segregation that they are capable of committing inhumane acts, since the physical and mental suffering imposed by the pain without correct analgesia is intense. In this regard, the failure of organizations to provide adequate service to people is exposed, due to color, culture, racial or ethnic origin^(26)^.

In addition to the above, living with the process of falling ill with SCD motivates men to find coping measures to deal with the disease and remain in the world of work. The adequacy to activities of daily living makes the man useful and valued, making them be seen and accepted socially with respect and recognized in the male role of invulnerability.

On the other hand, it is observed that falling ill with SCD reconfigures the male places in the universe of work and care, changing social, affective/relational and physical positions, giving rise to sensitivity, motivation and acceptance, improving coexistence with treatment diagnosis and compliance^(27)^.

Therefore, establishing actions and strategy becomes essential, since the economic difficulty is one of the main factors that significantly contributes to clinical variability and prognosis of people with SCD, with the groups mostly affected at the base of the social pyramid with low epidemiological and educational^(16)^.

From this perspective, the fact that SCD exposes individuals to situations of economic vulnerability, due to expenses with treatment and the cost of household expenses, arouses concerns in men, which makes them find strategies to continue working, either after retirement or in informality^(6)^.

In Brazil, with the emergence of public policies aimed specifically at this population, it is observed that, by facilitating medication dispensation, specialized care and provision of information to clarify the consequences of the disease, collaborate to appease the evolutionary process of the disease and meet the basic human needs of these individuals, as well as contribute to keeping this population active and productive^(28)^.

The family, especially mothers, play an important role in the lives of men with SCD and SCU, because culturally, in our society, women are assigned the role of caregiver. Thus, mothers represent financial, emotional and self-care guidance support, becoming relevant in men’s compliance with treatment as well as a strategic instrument for strengthening and learning skills, to make these individuals their own caregiver agents^(10)^.

From this perspective, nurses also emerge as an important agent to promote self-care and ensure that these men remain well to carry out activities of daily living, including work. Thus, nurses develop educational actions in the waiting rooms of outpatient clinics and offices, in community meetings, in individual care in offices, addressing aspects of the disease’s pathophysiology, main signs and symptoms, care with SCU, healthy habits, vaccination, which prevents injuries to health and promotes a better quality of life^(21,29)^.

Religion also emerged as a predictor of well-being, providing social and psychological support. Thus, the search for religious practices is one of the alternatives for coping with health problems. Attending church, saying prayers or listening to religious music appear as strategies that help to feel healthy and, sometimes, improve pain, in addition to being a space of refuge and social support^(28)^.

The limitation of this study lies in the fact that the results should not be generalized, as participants are limited to a restricted context and to a reality that may differ from other regions of the country. However, it is understood that the findings of this research can encourage the development of other more comprehensive studies, in addition to awakening health professionals’ interest to deepen their knowledge involving the care of people with SCD and SCU, enabling safe, effective and safe and humanized care for this population.

## CONCLUSIONS

The repercussions of SCD and SCU involve the biopsychosocial dimensions of individuals affected by these conditions, going through the pain and the health team’s frequent neglect in treating the manifestations of the disease with competence and dignity, especially in understanding the complexity and fully meeting the needs health of this population. Also noteworthy is the social issue involving SCD in men with low education, black, with altered body image and limited financial resources to provide for material needs. These characteristics demand the deconstruction of gender issues that negatively impact the evolution of the disease and treatment, the fight against structural racism and the need for the State to implement public policies that ensure material provision, especially of quality medications and treatments, to maintain the health of these men.

From the perspective of material provision and of feeling useful, productive and accepted in society, work occupies a relevant space in the lives of men with SCD and SCU, through which, many times, they forget that they have the disease and feel autonomous, occupying the important social role of providers of the home and of themselves. However, it was found that developing work activities is not a simple task, as many occupations can negatively alter the evolution of the disease and injury, as well as the acceptability of these men in the work environment.

In this regard, these men develop strategies to remain productive, highlighting the breaks in the workday to rest and lift the lower limbs, use of analgesics, often on their own to ease the pain, allocation of time during work to carry out the dressings, in addition, always showing confidence and readiness to develop work activities. Religion is highlighted as a strategy to remain hopeful and, therefore, confident in carrying out their work and their role in society. Furthermore, the family support network was evident, especially from the mother, to enable continuity of daily life activities and, in these, work.

This article intended to contribute to care practice in nursing and health, making the repercussions of SCD and SCU more evident in men’s daily lives, especially in relation to experiences related to the world of work. Also, it is expected that the results of this study will contribute to understanding the complexity involved in caring for men with this disease and the impacts on the lives of these individuals as well as favoring the creation and consolidation of public policies and actions of the Health Care Network targeted at this population.
